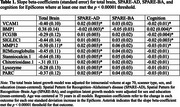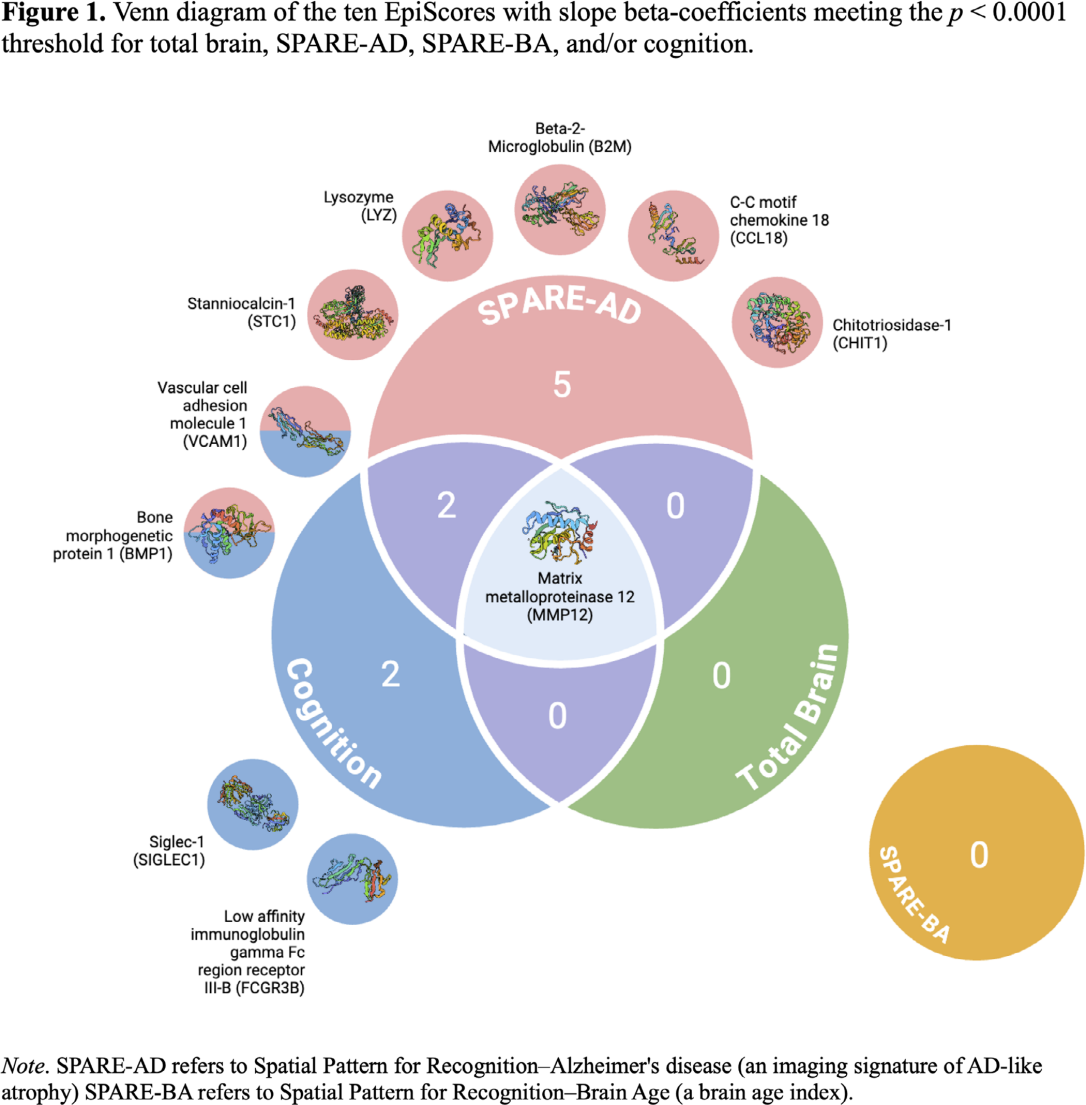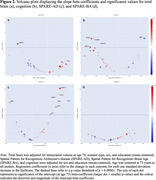# Epigenetic signatures of plasma protein abundance are associated with longitudinal brain and cognitive trajectories in older adults

**DOI:** 10.1002/alz70856_103678

**Published:** 2025-12-25

**Authors:** Shannon M. Drouin, Pei‐Lun Kuo, Cassandra O Blew, Michael R. Duggan, Cassandra M Joynes, Diefei Chen, Zenobia Moore, Guray Erus, Christos Davatzikos, Susan M. Resnick, Keenan A. Walker

**Affiliations:** ^1^ Laboratory of Behavioral Neuroscience, National Institute on Aging, Intramural Research Program, Baltimore, MD, USA; ^2^ Translational Gerontology Branch, National Institute on Aging, Intramural Research Program, Baltimore, MD, USA; ^3^ Department of Epidemiology, Bloomberg School of Public Health, Johns Hopkins University, Baltimore, MD, USA; ^4^ Centre for Biomedical Image Computing and Analytics, University of Pennsylvania, Philadelphia, PA, USA; ^5^ Department of Radiology, University of Pennsylvania, Philadelphia, PA, USA; ^6^ Laboratory of Behavioral Neuroscience, National Institute on Aging Intramural Research Program, National Institutes of Health, Baltimore, MD, USA

## Abstract

**Background:**

The biological processes underlying heterogeneous brain and cognitive trajectories in aging have been extensively informed through associations with the plasma proteome; however, circulating protein levels can be highly variable, susceptible to measurement error, and vulnerable to other fluctuations. Epigenetic approaches, such as those which proxy protein expression using DNA methylation (DNAm) measures, may represent more stable measures of circulating protein abundance and in turn be more robustly associated with brain aging and neurodegeneration‐related outcomes.

**Methods:**

Epigenetic (DNAm) data from a subsample of cognitively normal older adults from the Baltimore Longitudinal Study of Aging with longitudinal 3T MRI structural neuroimaging (*n* = 431; age=69.8 [12.1], up to 8 visits and 16.4 years of follow‐up) and longitudinal cognitive data (*n* = 681; age=73.1 [9.5], up to 8 visits, and 20.2 years of follow‐up) were used. We applied latent growth curve models (Mplus 8.2) to explore the association of 109 protein‐based EpiScores with longitudinal trajectories of (i) total brain volume, (ii) brain atrophy in regions vulnerable to Alzheimer's disease (SPARE‐AD), (iii) advance brain aging (SPARE‐BA), and (iv) cognition.

**Results:**

At a significance threshold of *p* < 0.00001, ten EpiScores were associated with slope of at least one tested outcome (Table 1, Figures 1‐2). Elevated scores on five (B2 Microglobulin, Stanniocalcin.1, Chitotriosidase.1, Lysozyme, PARC) EpiScores were uniquely associated with an increase in SPARE‐AD. Elevated scores on two (FCG3B, SIGLEC1) EpiScores were uniquely associated steeper cognition slope. An elevated VCAM EpiScore and a lower BMP EpiScore was associated with an increase in SPARE‐AD and decline in cognition. An elevated MMP12 EpiScore was associated with steeper decline in total brain volume and cognition, as well as increases in SPARE‐AD. Subsequent proteome‐wide analyses found EpiScore MMP12 (i) accounted for 22% of variation in concurrently measured plasma MMP12 (*n* = 186; rho=0.47; *p* < 0.0001) and (ii) correlated with 677 (of 7269) additional plasma proteins (*p* <0.001). Enrichment analyses of this proteomic signature implicated DNAm MMP12 in multiple disease‐relevant processes, including ephrin signaling, pro‐inflammatory response, and cytokine activity.

**Conclusions:**

Our analyses revealed several epigenetic indicators of circulating protein abundance (e.g., a DNAm measure of MMP12) to be strongly associated with trajectories of cognitive aging and MRI‐defined neurodegeneration.